# Co-infection of Oncogenic Human Papillomavirus 16 Genotype in Genital Warts: A Case Report

**DOI:** 10.7759/cureus.102200

**Published:** 2026-01-24

**Authors:** Ian M Santiago Velazquez, Hillary Mercado-Figueroa, Solimar Garcia Molina, Lillian V Rivera, Claudio Bernaschina-Bobadilla, Lynnette A Ruiz

**Affiliations:** 1 Medicine, Ponce Health Sciences University, Ponce, PRI; 2 Obstetrics and Gynecology, St. Luke’s Episcopal Medical Center, Ponce, PRI; 3 Public Health Sciences, Ponce Health Sciences University, Ponce, PRI; 4 Dermatology, Ponce Health Sciences University, Ponce, PRI; 5 Urology, St. Luke's Episcopal Medical Center, Ponce, PRI; 6 Basic Sciences, Ponce Health Sciences University, Ponce, PRI

**Keywords:** condyloma acuminata, high-risk human papillomavirus, hpv 16, hpv co-infection, hpv vaccination, human papillomavirus (hpv), koilocytosis, oncogenic, penile intraepithelial neoplasia, penile squamous cell carcinoma

## Abstract

Human papillomavirus (HPV) is globally one of the most common sexually transmitted infections (STIs). HPV 6 and 11, which are low-risk subtypes, are commonly associated with condyloma acuminatum. These benign lesions are typically described as papillary or cauliflower-like. We present the case of a 28-year-old uncircumcised male patient with these lesions on his foreskin. They were surgically removed, and upon histopathologic evaluation, the diagnosis of condyloma acuminatum was made. Real-time polymerase chain reaction (RT-PCR) was performed on the excised tissue for investigational purposes, and a high-risk HPV (hr HPV) strain was identified. Testing of this specimen did not influence the immediate clinical management in this case. Even though HPV subtype 16 is not generally associated with genital warts, its detection in an otherwise benign lesion illustrates the limitations of relying on clinical appearance alone and suggests the possibility of evaluating and managing these lesions on a case-by-case approach. This case highlights the role of histopathologic evaluation and selective molecular testing in carefully selected patients and clinical scenarios, such as persistent lesions and atypical appearance. As this is a single case report, the findings depicted here cannot be generalized and do not support changes to current screening or management guidelines. HPV vaccination remains the most effective method for preventing HPV-related disease.

## Introduction

Human papillomavirus (HPV) is one of the most common pathogens that is typically associated with sexually transmitted diseases (STDs) on a worldwide scale, and the incidence of HPV infection is rising, especially among sexually active individuals [1]. HPV genotypes are broadly categorized into low- and high-risk types based on their oncogenic potential. HPV types 6 and 11 are responsible for low-risk anogenital lesions, such as condyloma acuminatum, which can be diagnosed by careful observation of their distinctive papillary appearance [2]. In contrast, oncogenic or high-risk HPV (hr HPV) genotypes, including types 16 and 18, are associated with malignant transformation and are implicated in several cancers, including penile cancer.

In a study by Freire et al. (2014), 59% of patients were found to have co-infection with different HPV types, including hr HPV genotypes such as 16 and 18 [3]. Although genital warts are considered benign lesions, detection of cancer-associated HPV types within these lesions has been reported. Identification of HPV 16 in benign-appearing genital warts is uncommon because such lesions are typically attributed to low-risk HPV types and are usually treated with destructive therapies without prior knowledge of histopathology or molecular testing [4]. As a result, oncogenic HPV infection may remain undetected, particularly in men, for whom routine hr HPV testing is not part of preventive healthcare visits.

We present the case of a 28-year-old male patient with genital warts in the foreskin area that were excised surgically, with a pathological diagnosis confirming condyloma acuminatum. For research purposes, we performed a polymerase chain reaction (PCR) test for hr-HPV genotypes, which revealed the presence of HPV-16. This finding is clinically significant because it exemplifies that oncogenic HPV genotypes can be present in benign-appearing genital lesions that would not otherwise undergo molecular evaluation. Therefore, the objective of this clinical case is to highlight the importance of histopathological examination and consideration of hr HPV testing in excised genital warts, as early identification of oncogenic HPV may represent a potential risk factor for penile cancer and inform future preventive strategies [4].

## Case presentation

We present the case of a 28-year-old uncircumcised male patient who sought evaluation for a cauliflower-like lesion with flesh-colored bumps on his foreskin. This was consistent with condyloma acuminatum. At presentation, details about symptom duration or prior treatments were unavailable. There were no signs of malignancy. Surgery was performed to confirm the diagnosis and provide treatment, as described below.

Under an appropriate sterile environment and with local anesthesia, the foreskin was retracted to expose the glans penis. Adhesions were lysed. A circumferential incision at the coronal sulcus was performed. The foreskin was excised using standard surgical techniques. During the procedure, hemostasis was accomplished using electrocautery and absorbable sutures. The skin and mucosal edges were approximated with fine absorbable sutures to support healing and optimize cosmetic results. Later, a sterile dressing was applied. After the procedure, the patient was informed about hygiene measures and was scheduled for follow-up to assess wound healing. The excised tissue specimen was submitted for histopathological and molecular analysis.

According to the pathology report, the specimen labeled as “prepuce” consisted of a portion of foreskin measuring 3 × 2.3 cm and displayed several verrucous lesions ranging in size from 0.1 to 0.5 cm; a representative section was submitted in one cassette. Additionally, the specimen labeled as condylomas consisted of several pinkish-tan, soft-tissue fragments measuring 1 × 0.8 × 0.3 cm and was submitted in a single cassette.

HPV genotyping was performed on the preserved foreskin tissue for research and educational purposes. This did not influence the patient's immediate clinical management. Real-time-PCR (RT-PCR) testing was performed at a private laboratory specializing in molecular diagnostics. The assay screened for 14 high-risk HPV genotypes. These included types 16, 18, 31, 33, 35, 39, 45, 51, 52, 56, 58, 59, 66, and 68. RT-PCR analysis detected HPV 16, a high-risk genotype associated with malignant potential at anogenital and/or oropharyngeal sites. Standard RT-PCR protocols were used. Results were extracted using BrioQuery version 6.6 (Brio Software Inc., Santa Clara, CA, USA) and recorded in Microsoft Excel (Microsoft Corporation, Redmond, WA, USA) for analysis.

**Figure 1:**
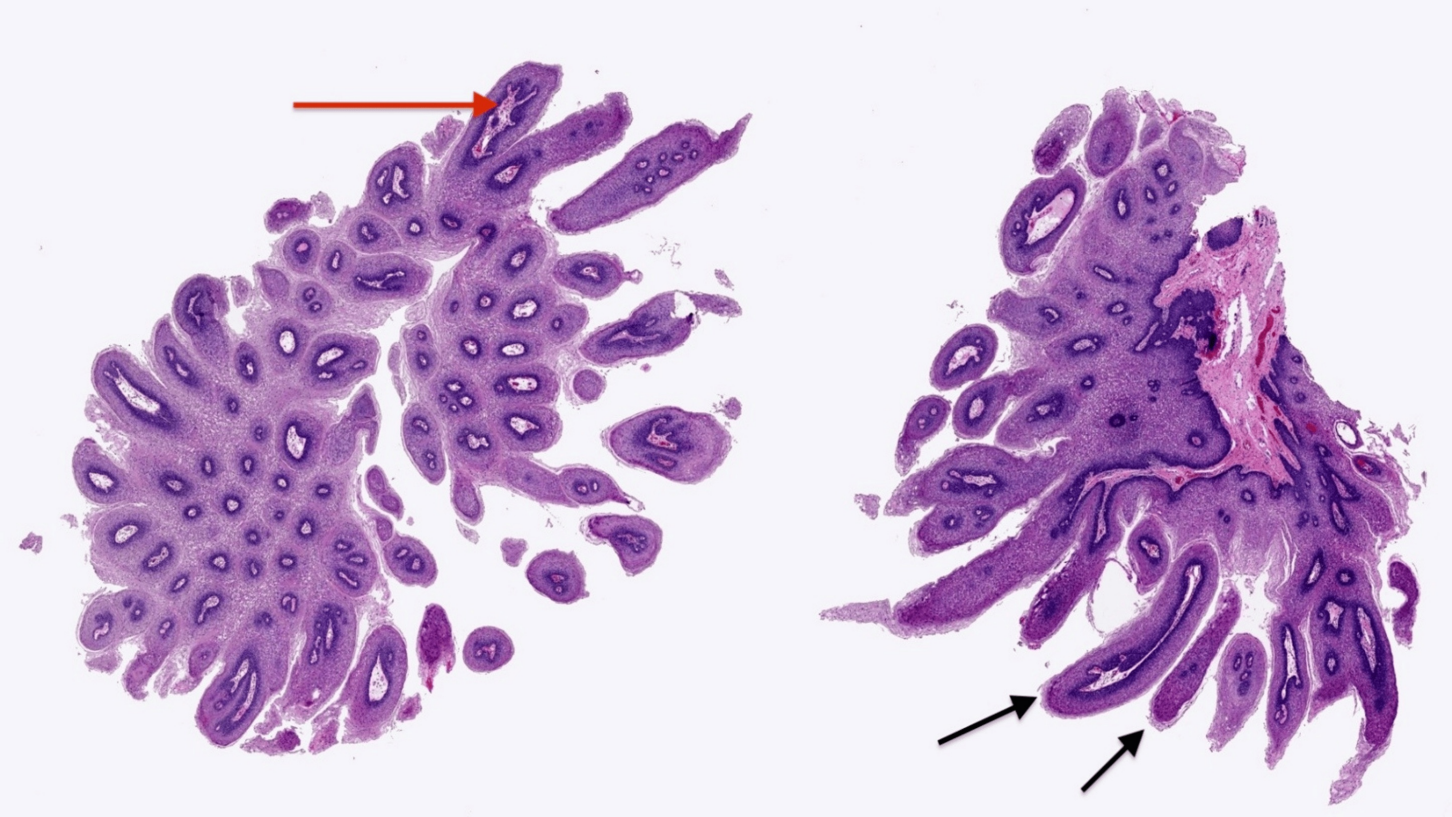
Papillomatosis with finger-like epithelial projections and fibrovascular cores. Hematoxylin and eosin–stained section demonstrating papillomatosis with multiple frond-like, finger-shaped epithelial projections (black arrows) containing central fibrovascular cores (red arrow), a characteristic architectural feature supporting the diagnosis of condyloma acuminatum. Low-power view. Image reproduced with courtesy of PathologyOutlines.com, contributed by Kavita Umrau, MBBS, and Bin Xu, MD, PhD [5].

**Figure 2:**
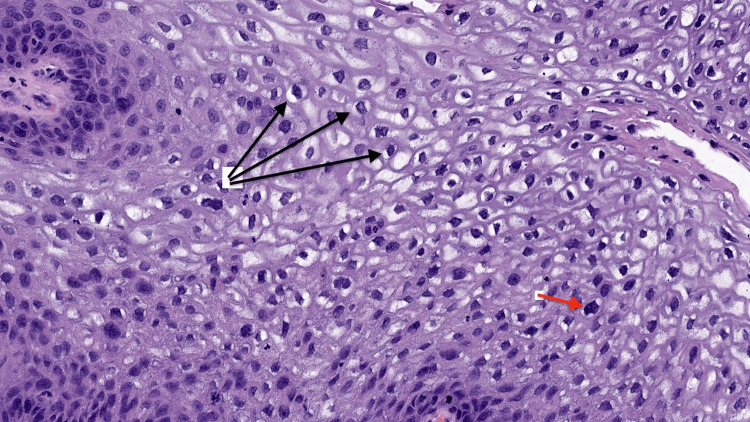
Koilocytosis demonstrating human papillomavirus-associated cytopathic changes. Hematoxylin and eosin-stained section demonstrating koilocytosis within the superficial epithelial layers. Koilocytes with perinuclear halos (black arrows) and nuclear membrane irregularities (red arrow), with occasional binucleation, are shown, hallmarks of human papillomavirus infection that support the diagnosis of condyloma acuminatum. Image reproduced with courtesy of PathologyOutlines.com, contributed by Kavita Umrau, MBBS, and Bin Xu, MD, PhD [5].

Histopathologic examination confirmed the diagnosis of condyloma acuminatum (genital warts). Classic features were observed, including papillomatosis (finger-like skin projections) and koilocytosis (cells with changes typical of HPV infection). These are illustrated in Figures 1, 2 [5].

## Discussion

HPV is one of the most common sexually transmitted infections (STIs) worldwide, with over 200 types identified [6]. Hr HPV types such as 16 and 18 are linked to cancers of the cervix, penis, anus, and oropharynx, while low-risk types like HPV 6 and 11 are most often responsible for genital warts and are not described as oncogenic [7]. Co-infection with hr HPV strains, especially HPV 16, has been associated with an increased risk of developing cancers in the genital and oropharyngeal regions due to their oncogenic potential and role in malignant transformation at these anatomical sites [8].

Most HPV infections are transitory and asymptomatic, with a high number of them clearing spontaneously within one to two years. Still, persistent infections with hr HPV subtypes such as HPV 16 and 18 can lead to the development of precancerous lesions and, over time, malignancy in the anogenital and/or oropharyngeal regions [9]. In men, genital warts are commonly caused by HPV types 6 and 11, which account for roughly 90% of cases, and these warts are benign and not associated with cancer. The median time for this infection to clear in men has been estimated at around six months, and 75% of these infections are cleared within one year [10]. Even though HPV 16 is not commonly associated with genital warts, its detection at these sites frequently indicates co-infection with low-risk subtypes like HPV 6 and 11, which particularly occurs in immunosuppressed individuals. Its presence is clinically noteworthy because of its high oncogenic potential [10]. The foreskin's moist environment and thinner skin make it more vulnerable to infection, which may lead to the increased incidence of penile cancer in uncircumcised men. Hr HPV has been detected in 42% to 48% of PeSCC and PeIN, with HPV 16 accounting for over 60% of HPV-positive cases [11]. The expression of p16, which is a surrogate marker for oncogenic HPV activity, is frequently found in these lesions, providing additional evidence for the link between hr HPV and penile cancer [12].

The United States Centers for Disease Control and Prevention (CDC) recommends that genital warts be diagnosed primarily by physical examination, with tissue testing reserved for atypical, suspicious, or persistent lesions. Even so, detection of hr HPV in typical-appearing warts supports selective use of pathology and molecular testing in specific clinical scenarios. The CDC does not recommend routine HPV testing for men, as there are no approved screening tests, and most infections are subclinical. Nevertheless, a selective histopathologic and molecular work-up may be considered for individuals with persistent or suspicious lesions, as these lesions may be associated with underlying risk factors for developing penile squamous cell carcinoma (PeSCC) and penile intraepithelial neoplasia (PeIN) [13]. This was highlighted in the case of a 28-year-old otherwise healthy male patient who presented to the clinic with a wart-like penile lesion; biopsy confirmed it to be condyloma acuminatum, with hr HPV 16 detected by molecular testing. This case documents an uncommon finding of hr HPV detection in a histologically confirmed condyloma acuminatum and draws attention to the potential value of biopsying suspicious or persistent lesions in select clinical contexts.

Male patients in Puerto Rico need to be informed about HPV vaccination and the possible dangers related to hr HPV co-infections. HPV is one of the most common sexually transmitted infections worldwide and remains a possible source of several cancers in men, including PeIN, PeSCC, anal, and/or oropharyngeal cancers. A recent study in Puerto Rico found that over a third of young women had high-risk HPV. In more than 85% of those cases, the types were not HPV-16 or HPV-18 [14]. This highlights the potential risk for co-infections with multiple oncogenic strains. As we know, men are not routinely screened for HPV like women. However, they play a central role in transmission and are also at risk of developing HPV-related cancers like PeIN and PeSCC. Vaccination offers critical protection by not only reducing the spread of high-risk HPV types but also lowering individual cancer risk. Early vaccination, ideally before the onset of sexual activity, along with increased public awareness about persistent HPV infections, is essential to reducing the overall burden of HPV-related diseases in Puerto Rico.

Early identification of hr HPV may help inform closer follow-up in select clinical scenarios. Vaccination is the most effective strategy for preventing HPV-related diseases, including cancer. Current guidelines do not recommend routine screening for STIs such as chlamydia, HPV, and gonorrhea in asymptomatic men due to insufficient evidence; however, men continue to play a role in transmission and have rising infection rates [15,16]. It has been shown that the rate of chlamydia among men increased by 32.1% from 2015. Despite this data, evidence persists as insufficient to support routine screening in men [15,16].

HPV vaccination in men remains underutilized, despite its role in preventing cancers and other health complications. Increasing access to HPV vaccination and integrating co-testing strategies can help close current prevention gaps [15]. This is especially important considering the overlapping routes of infection and consequences of STIs. Syphilis trends also reflect the need for more male-focused STI prevention. Rates of primary and secondary syphilis increased from 2.1 cases per 100,000 in 2000 to 2001 to 11.9 in 2019, with men accounting for 83% of cases [17]. The United States Preventive Services Task Force recommends screening for those at increased risk [17]. Comprehensive human immunodeficiency virus care highlights similar concerns, as persons with the virus are living longer due to antiretroviral therapy, which shifts the focus to long-term health, including cancer screening, vaccination, and STI prevention [18]. Stigma-free, patient-focused care is necessary to maintain regular involvement and address comorbidities [18].

The results show a gap in how we approach STI prevention, especially when it comes to men. Infections such as HPV, chlamydia, gonorrhea, and syphilis are on the rise, but many prevention efforts are not reaching or being followed by the population. Men are a key part of how these infections spread and face serious risks, including cancers of the penis, anus, and throat. Even so, routine HPV screening for men is not generally recommended. Because of this, infections such as HPV, paired with high-risk subtypes, could have serious complications (e.g., PeIN or PeSCC) and might go unnoticed, especially when someone has more than one infection or a weakened immune system. In Puerto Rico, the burden of penile cancer mortality among men is significantly higher than in other U.S. racial and ethnic groups and up to three times higher than in non-Hispanic White men. This elevated risk prevails across most age groups and is associated with social determinants, including low levels of education and socioeconomic status [19].

Dealing with these gaps requires expanding HPV vaccination to boys and young men, carrying out focused screening for those at higher risk, and improving education around STI transmission and complications. Because STIs are transmitted in similar ways and share symptoms, we need to develop forms of prevention for sexually active individuals. Vaccination and screening based on risk can aid in lowering the impact of HPV and other STIs in places like Puerto Rico, where access to care, awareness, and culturally sensitive services persist as major obstacles.

As a single case report, our observation is subject to inherent limitations. It cannot be used to estimate the prevalence of high-risk HPV co-infection in genital warts, confirm a causal relationship, or quantify future risk of PeIN or PeSCC. Rather, it serves to document an uncommon presentation and to generate hypotheses for future investigation.

## Conclusions

In this case, high-risk HPV type 16 was found in a lesion that appeared benign and was consistent with a genital wart. This case shows that oncogenic HPV types can be present even when lesions look characteristic and do not raise immediate concern. Condyloma acuminatum is usually linked to low-risk HPV types and is often treated without further testing. However, this case suggests that additional pathologic review and selective molecular testing may be considered in atypical presentations. Detection of HPV 16 in this lesion demonstrates the limits of relying on appearance alone. In some cases, evaluation may benefit from considering lesion behavior over time, histologic findings, and patient-specific risk factors together. Routine screening for hr HPV in men is not recommended, but clinical judgment may be used when deciding whether further evaluation is appropriate for selected lesions.

Because this report describes a single patient, the findings cannot be generalized and should not guide screening practices. Larger studies are needed to determine how often hr HPV is found in benign-appearing genital warts and its clinical significance. HPV vaccination remains the most effective strategy for preventing HPV-related disease, particularly for populations such as Puerto Rican men, who have a higher penile cancer risk.
